# Attention for Emotion—How Young Adults With Neurodevelopmental Disorders Look at Facial Expressions of Affect

**DOI:** 10.3389/fpsyt.2022.842896

**Published:** 2022-06-15

**Authors:** Jana Bretthauer, Daniela Canu, Ulf Thiemann, Christian Fleischhaker, Heike Brauner, Katharina Müller, Nikolaos Smyrnis, Monica Biscaldi, Stephan Bender, Christoph Klein

**Affiliations:** ^1^Department of Child and Adolescent Psychiatry, Medical Faculty, University of Cologne, Cologne, Germany; ^2^Clinic for Child and Adolescent Psychiatry, Psychotherapy and Psychosomatics, Faculty of Medicine, Medical Center, University of Freiburg, Freiburg im Breisgau, Germany; ^3^Clinic for Psychiatry, Psychosomatics und Psychotherapy in Children and Adolescents, LVR Hospital, Bonn, Germany; ^4^Kinder- und Jugendwohnheim Leppermühle, Buseck, Germany; ^5^Second Department of Psychiatry, National and Kapodistrian University of Athens, Medical School, University General Hospital “Attikon”, Athens, Greece

**Keywords:** Autism Spectrum Disorder, ADHD, schizophrenia, emotional faces, eye movements (EM)

## Abstract

While Autism Spectrum Disorder (ASD), Attention-Deficit/Hyperactivity Disorder (ADHD) and Schizophrenia (SCZ) differ in many clinically relevant features such as symptomatology and course, they may also share genetic underpinnings, affective problems, deviancies in social interactions, and are all characterized by some kind of cognitive impairment. This situation calls for a joint investigation of the specifics of cognitive (dys-)functions of the three disorders. Such endeavor should focus, among other domains, on the inter-section of processing cognitive, affective and social information that is crucial in effective real-life interactions and can be accomplished when attentional preferences for human facial expressions of emotions is studied. To that end, attention to facial expressions of basic emotions was examined in young adults with ASD, ADHD, or SCZ in the present study. The three clinical groups were compared with an age-matched group of typically-developing participants (TD) during the free contemplation of five different facial emotions presented simultaneously, by varying identities, through the registration of eye movements. We showed, that dwell times and fixation counts differed for the different emotions in TD and in a highly similar way in ADHD. Patients with ASD differed from TD by showing a stronger differentiation between emotions and partially different attentional preferences. In contrast, the SCZ group showed an overall more restricted scanning behavior and a lack of differentiation between emotions. The ADHD group, showed an emotion-specific gazing pattern that was highly similar to that of controls. Thus, by analyzing eye movements, we were able to differentiate three different viewing patterns that allowed us to distinguish between the three clinical groups. This outcome suggests that attention for emotion may *not* tap into common pathophysiological processes and argues for a multi-dimensional approach to the grouping of disorders with neurodevelopmental etiology.

## Introduction

According to DSM-5 (1), Autism Spectrum Disorder (ASD), and Attention-Deficit/Hyperactivity Disorder (ADHD) both belong to the group of “Neurodevelopmental Disorders” (NDD). This group includes a variety of impairments thought to involve a disorder of brain development (2). Main characteristics include genetic influences, multi-factorial etiologies, onset in childhood, prior to puberty, and a steady clinical course despite developmental changes. Developmental impairments also play a role in Schizophrenia (SCZ) and consequently this disorder is thought to have an etiology of neurodevelopmental abnormalities (3). By contrast to ASD and ADHD, SCZ has been categorized by the DSM-5 in the separate group of “Schizophrenia Spectrum and Other Psychotic Disorders” (SSD) (1).

ASD, ADHD, and SCZ differ in important aspects such as symptomatology and especially their developmental course: ASD onsets in infancy and does not improve much during development, whereas ADHD onsets around the age of 7 years and may show substantial improvements during adolescents (in particular, regarding hyperactivity). SCZ by contrast onsets in late adolescence or young adulthood and shows a variety of courses. While there are multiple differences, these disorders show commonalities in other important domains like deficits in cognitive abilities and social interaction (4, 5). Furthermore, due to genetic overlap between ASD, ADHD, and SCZ and other commonalities including developmental delays, motor deviations, higher incidence in males, frequent comorbidity, and common environmental risk factors, Owen and O'Donovan (6) have proposed to group these disorders (and intellectual disability and bipolar disorder) under the neurodevelopmental continuum model, a further development of earlier neuro-developmental models of SCZ [e.g., (3)].

Such models call for the joint investigation of these disorders to identify potential common or distinct pathophysiological mechanisms, which would, in turn, speak to nosology and clinical practice. A so called transdiagnostic approach finds support from neuroimaging results. In a recent review, Hoogman et al. (7) conclude that subcortical structures are affected in a similar way in ASD and ADHD, especially in volume. Cortical analyses showed specific differences but also overlaps especially in cortical thickness for ASD and ADHD. Consequently, a joint consideration of disorders with common neurobiological aspects seems reasonable.

Such joint investigation of ASD, ADHD and SCZ has received little attention in previous research [e.g., (8–10)], and the present study is one of the first to consider the cognitive alterations of ASD, ADHD, and SCZ in simultaneous comparison, focusing on attention for facial emotional expressions as a requirement of succeeding social interactions.

Processing the facial expressions of emotions is a special skill in non-verbal communication and indispensable for effective social interactions (11, 12). Most studies that address emotional facial expressions work with the concept of basic emotions, which are defined as facial expressions that can be observed across cultures (13) and include happiness, surprise, fear, anger, disgust, and sadness. These emotions are considered as universally expressed and innately decoded and recognized by typically-developed subjects, already early in childhood (14, 15).

Previous studies have shown differences in the accuracy of *emotion recognition* (ER) between these emotions, with happiness being easier recognized than fear, surprise or anger (16–18), whereas fearful and surprised facial expressions can be confused more easily (19–21).

Studies examining emotion recognition in children and adolescents with ASD, ADHD, or SCZ showed overall inconclusive results. Some studies showed that the recognition of basic emotions in those psychiatric disorders seems to be similar to that of neuro-typical controls [ASD: (22–26); ADHD: (27, 28); SCZ: (29–31)], while other have revealed recognition difficulties [ASD: (23, 32, 33); ADHD: (34–36); SCZ: (37–39)]. The inconsistency of results can be attributed to various methodological aspects (e.g., complexity of the task, time pressure, different stimuli and dependent variables) as well as sample heterogeneity [ASD: (40); ADHD: (41); SCZ: (42)]. For example some subgroups like high functioning and older patients with autism appear to employ compensatory mechanism in basic ER (22, 30, 40, 43, 44). Furthermore, attentional distractibility has been shown by Berggren et al. (45) to influence ER performance in ASD and ADHD. In that study ER problems did not show universally for ASD, and ADHD performance was little different from that of TD. This argues for abnormalities in central executive functions rather than in specific emotion recognition in participants with ADHD (45). Despite the mixed results, it may be assumed that patients with ASD, ADHD and SCZ can recognize at least basic emotional expressions at the simplest level from photos of faces to a comparable extent as healthy control subjects.

Regarding the method of investigation, eye tracking can provide important information about exploration behavior and the accompanying cognitive processes involved in emotion processing (46, 47). Eye movements and fixations can be recorded, by which the active process of seeing is characterized (48). Thus, by recording directed visual attention it is possible to map how subjects explore and reconstruct their visual environment.

To give examples, Green et al. (47) showed increased numbers of fixations for the facial expressions of anger and fear in healthy controls, which they explained by an increased vigilance related to socially threatening stimuli, allowing for a faster and adaptive behavior of the observer. In contrast, Mühlenbeck et al. (49), who looked at fixation times in healthy subjects for different emotional faces (fear, anger, happy, neutral), showed longer fixation times for fearful and shorter ones for angry faces, which argues against a general bias toward negative emotions. Accordingly, varying viewing paths and times of facial expressions can be observed (50). Herbold (12) also found longer viewing times and fixation counts for fearful faces compared to happy, angry, sad, and neutral ones as well as for surprised faces compared to joyful and neutral ones. Such studies therefore suggest that different emotions may cause differences in how faces are viewed, thus pointing to a different orientation of *attention for emotion*, or *attentional preferences*.

Findings from studies investigating eye movements during emotional face processing show abnormalities in face viewing in several psychiatric disorders, including ASD, ADHD, and SCZ, which could contribute to difficulties in complex emotion recognition and thus in social interactions.

In *autism*, abnormalities are shown in different viewing patterns for different emotions with mostly the same accuracy in emotion recognition as in control subjects is seen (51–54). Król and Król (52) examined eye movements in subjects with autism and typically developed subjects during an emotion recognition task in which photographs of faces were to be assigned emotions. Here, the subjects with autism achieved an accuracy of 85%, which was only slightly lower and comparable to that of controls (92%). There were no differences in total fixation number or fixation duration between ASD and TD in any of the tasks used (e.g., emotion recognition task, free viewing task). The authors, however, did not examine further differences between different emotions expressed. Likewise, typical recognition of facial expressions of the six basic emotions was reported by Tang et al. (53). Furthermore, the subjects with autism of that study showed longer fixation times for non-social areas when viewing social scenes. There were, however, no differences in viewing different facial and body areas between groups. These results demonstrate atypical visual processing and prioritizing of social stimuli by individuals with autism with comparable behavioral performance.

However, while it seems that individuals with autism potentially employ altered but functionally preserved processing strategies in emotion recognition (40, 54), some studies examining emotional preferences as revealed by eye fixations have reported an attentional bias away from distressing stimuli such as angry or fearful stimuli in children with ASD (51, 55). For example, García-Blanco et al. (51) found an attentional bias away from angry faces in individuals with autism compared to a control group, but no differences for happy or sad faces. This attentional bias was correlated with higher scores on social communication deficit. Matsuda et al. (56) asked children with ASD to look at photos of individual emotional faces (angry, happy, neutral, sad, surprised) and found no differences in gaze behavior between ASD and TD. However, there was a slight inverse relationship between autistic symptomatology and looking duration for angry faces. In addition to these studies, there are also studies that showed a bias toward looking at distressing stimuli in ASD (51, 57). A recent study by Bochet et al. (58) examined emotional face processing *via* eye-tracking in children with autism and an age-matched TD group. They showed pairs of faces, a neutral face paired with an emotional face, of the same identity. They observed different exploration behavior between ASD and TD. ASD made fewer fixations regarding the emotional faces, from which the authors concluded that emotional faces were less interesting to ASD. A meta-analysis summarizing the results regarding an attentional bias in ASD showed a small but significant and specific effect for a bias toward threatening faces under certain conditions such as line-drawings of emotional faces or in comparison to happy faces (59). Other moderators such as stimulus presentation, response format, reference face, stimulus type, and age had an influence on the strength of this bias. Overall, however, it appears that atypical emotional preferences in ASD have so far not been replicated consistently across studies, necessitating further investigation.

Processing of emotional faces has been studied considerably less in participants with *ADHD* especially in combination with eye movement measurements but suggests typical performance in this group. Schwenck et al. (27), for instance, showed patients with ADHD and control subjects film clips in which a neutral face develops into one of different basic emotions (happy, sad, disgust, fear, anger). They found no differences in reaction times or recognition performance between the groups. Similar results were obtained by Serrano et al. (60), who reported overall typical viewing patterns in participants with ADHD when looking at photos of faces showing the six basic emotions and neutral control faces. While emotion processing *per se* seems to be intact in ADHD, the attentional problems in this group may render important emotional cues unattended (34, 60). In a study by Ahmadi and Judi (61), who looked at viewing preferences during the presentation of emotional face pairs (negative-neutral), there were no differences between children with ADHD and TD subjects regarding the number of first fixations on the emotional expressions. Pishyareh et al. (62) are also one of the few to use eye tracking to study visual exploration of emotional stimuli in children with ADHD. They were able to show that patients with ADHD spend less time looking at pleasant pictures than control subjects when presented with unpleasant or neutral pictures simultaneously. These results provide some evidence that attention for emotion may differ between children with and without ADHD. Again, more data are needed here to draw firmer conclusions.

A large number of studies have shown impaired attentional distribution in participants with *schizophrenia* compared to healthy control subjects, especially when viewing faces compared to other complex stimuli (63–66). Specifically, SCZ participants often show restricted, centrally focused exploration behavior (67, 68) that can be described by reduced scan path length, fewer fixations (69) and shorter fixation durations for faces (70). Despite such constrained exploration patterns, emotion recognition as such seems to be preserved in those with SCZ (71). Importantly, this deviant exploration behavior is also found independently of the expressed emotions of the faces being viewed, suggesting a face-specific and perceptual processing deficit rather than emotion-specific processing deviations or difficulties (72, 73). In line with this reasoning, Asgharpour et al. (69) examined visual attention in SCZ by measuring eye movements during the viewing of pairs of faces consisting of an emotional and a neutral face. They found that SCZ patients showed fewer fixations on faces, regardless of the presence or absence of a displayed emotion.

Importantly, in previous eye movement studies on facial emotion processing, fixation parameters (duration, counts) were typically determined for tasks in which emotions were presented either individually or in pairs (12, 22, 51, 54). Such reduced “choice” of different emotions available for contemplation constrains participants' ability to express attentional preferences in their viewing patterns. This raises the question which emotions are preferred and focused in situations when *several* emotional faces are presented simultaneously. García-Blanco et al. (74), for instance, presented four emotional images (happy, neutral, sad, threatening) simultaneously to participants with bipolar disorder (BD) and recorded eye movements to examine the distribution of attention. They found that patients with bipolar disorder showed increased attention to threatening images compared to healthy individuals, which they interpreted as a vulnerability marker in BD.

To the best of our knowledge, attention for emotional faces has not yet been investigated by presenting several faces simultaneously in psychiatric disorders with profound deficits in social interaction using eye movement recordings. Therefore, this study will examine this most basic level of emotion processing in a direct comparison. Based on these approaches and results, the aim of this study is to look at the exploration behavior of different emotional faces simultaneously in ASD, ADHD, and SCZ and to investigate differences and similarities compared to a healthy control group. (1) We hypothesized that emotion-specific differences in dwell time and fixation count exist for the presented emotions of fear, surprise, happiness, anger and neutral faces. (2) Furthermore emotion-specific group differences in dwell time and fixations are expected to exist between the autism group and the control group, due to the described deviant visual emotion processing in ASD. (3) Since the ADHD group predominantly showed similar visual exploration as healthy control subjects, no group differences are expected. (4) Regarding the clinical groups, compared to a healthy control group, patients with SCZ are expected to show a constrained visual exploration behavior, expressed in a shorter dwell time and fewer fixations for the presented faces, without differentiation of the emotions shown.

## Methods

### Participants

The final sample consisted of four groups of participants: *N* = 38 individuals were patients with Attention Deficit/Hyperactivity Disorder (ADHD), *N* = 28 with Autism Spectrum Disorder (ASD), *N* = 21 with Schizophrenia (SCZ) and *N* = 41 individuals were typically-developing participants (TD). All participants had normal or corrected-to-normal vision and no one had a diagnosis of epilepsy or another neurological disease. The groups did not differ significantly in age (see Table 1).

**Table 1:** Group characteristics.

**Variable**	**SCZ**	**ADHD**	**ASD**	**TD**	***F*** **_3, 124_**	***p***
*N*	21	38	28	41		
Mean age (in years) ± SD	19.8 ± 1.7	19.0 ± 2.2	19.4 ± 2.2	18.9 ± 2.1	1.068	0.365
Gender (% female)	29	42	4	63	-	-
SPM (% correct)	47.2 ± 27.2	59.1 ± 23.7	70.2 ± 20.5	68.0 ± 15.8	5.878	0.001

*SD, standard deviation; SCZ, schizophrenia; SPM, standard progressive matrices*.

Patients with a diagnosis of ADHD or ASD were recruited from the out-patient population of the Clinic for Child and Adolescent Psychiatry, Psychotherapy, and Psychosomatics of the Medical Center, University of Freiburg. Participants with SCZ were recruited from the rehabilitation center “Kinder- und Jugendwohnheim Leppermühle” (Buseck, D) and had received a diagnosis of schizophrenia, schizophreniform or schizoaffective disorder. The typically-developing participants of the healthy control group were recruited through the project database of the department and by posting announcements.

All TD participants were asked for their medical history during a phone screening at the time of enrollment. Any present or past personal or family history of psychiatric disorder (and/or any neurologic condition) was an exclusion criterion. All participants in the clinical groups had received a prior clinical diagnosis. Given the high degree of co-morbidity of ASD and ADHD and the very high degree of all kinds of co-morbidity in all neurodevelopmental disorders, we excluded patients with both ASD and ADHD and also those with a co-morbid substance use disorder. Furthermore, we excluded one patient with SCZ who also had an ADHD diagnosis. Diagnoses of ADHD were confirmed by the German version of the Conners' parent and teacher rating scale (75), interviews with parents and children, and behavioral observations. Diagnoses of ASD were confirmed using the Autism Diagnostic Observation Schedule [ADOS; German version, (76)] and the Autism Diagnostic Interview-Revised [ADI-R; German version, (77)]. To assess the specificity and severity of ADHD and ASD symptoms, plus their absence in the TD group the Social Responsiveness Scale [SRS; German version, (78)] and the Conners' Self and Parent Rating Scale were used. Participants who were taking methylphenidate medication were therefore asked to pause medication 24 h before and during participation. Regarding the SCZ group, all participants of our study had previously taken part in a follow-up study (catamnestic) during which various established research diagnostic measures had been administered, including IRAOS (“Interview for the Retrospective Assessment of the Onset and Course of Schizophrenia and other Psychoses”), CGI/GAF (“Clinical Global Impressions,” “Global Assessment of Functioning Scale”), GAS (“Global Assessment Scale”), SAPS/ SANS (“Scale for the Assessment of Positive/Negative Symptoms”), BPRS (“Brief Psychiatric Rating Scale”). Clinical patient records for all participants with SCZ were scrutinized to obtain information about current medication and current previous co-morbid diagnoses. They were treated with (antipsychotic) medication as follows: *N* = 11 Clozapine 150–400 mg, *N* = 5 Aripiprazol 2.5–20 mg; *N* = 3 Olanzapine 5–10 mg; *N* = 3 Quetiapin 300–400 mg; *N* = 6 Dipimperon 20–60 mg; *N* = 3 Risperdal 0.5–4.5 mg; *N* = 4 Venlafaxine 150–225 mg; *N* = 3 Fluoxetin 20 mg; *N* = 3 Escitalopram 20 mg.

IQ was tested using the CFT 20-R (79) for participants in the ADHD, ASD, and TD group and the Wechsler Intelligence Test [WISC-IV for children, WAIS-IV for adults; German version, (80)] for participants in the schizophrenia group. In addition, a 9-item short version of the Raven Standard Progressive Matrices [RSPM, (81)], correlating with the 60-item version by *r* = 0.98 (82), was conducted during the ocular-motor session. Groups differed significantly in IQ (see Table 1).

Ethical approval to the study was given by the Ethics Committee of the Albert Ludwigs-University Freiburg (EK124/17). All adult participants signed a consent form, for minors both from parents and minors informed written consent was obtained.

### Stimuli

**Figure 1:**
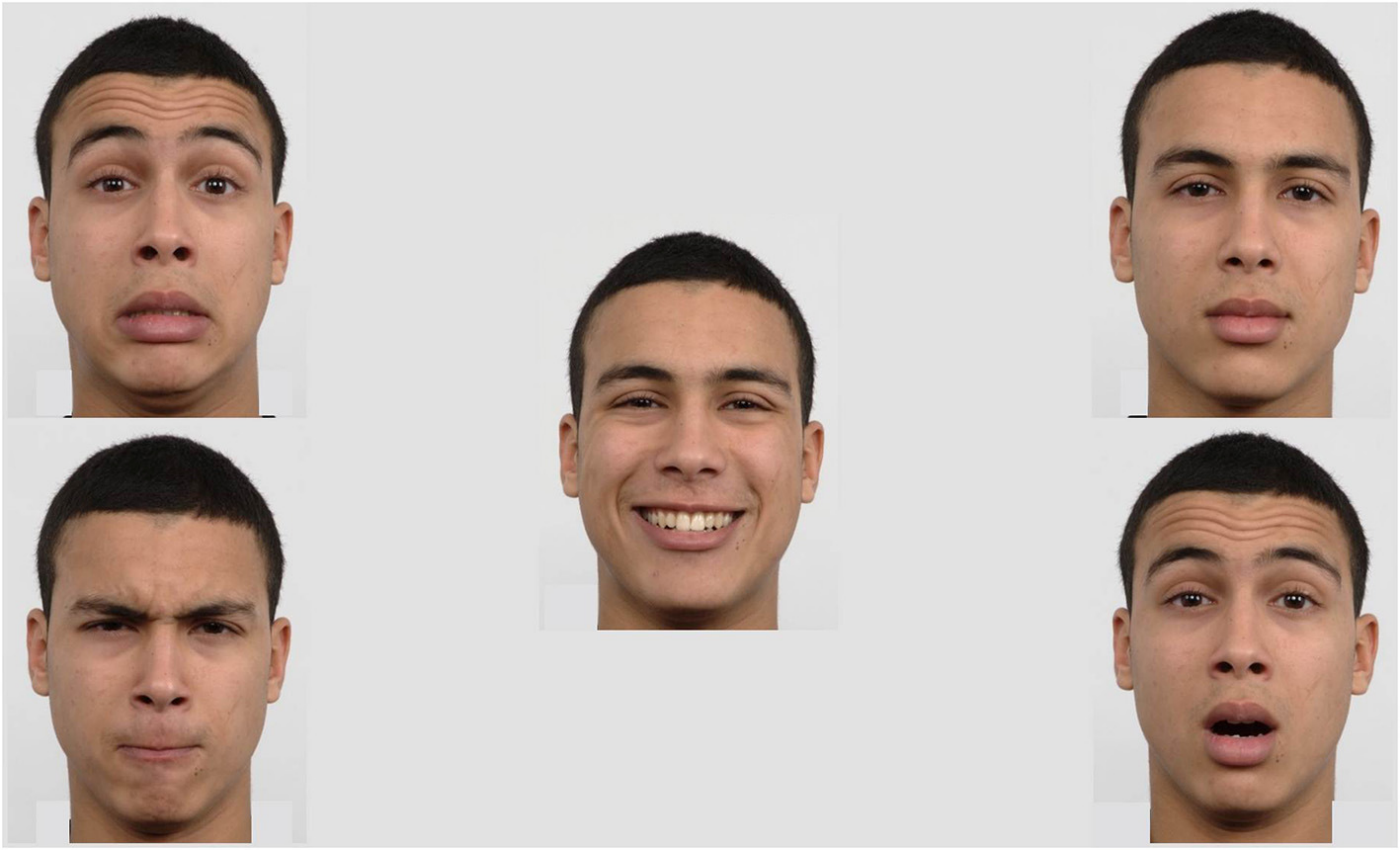
Exemplary stimulus: The five emotional expressions (happy, neutral, surprised, angry, and scared) of one identity were presented simultaneously on five different positions (center, top right, bottom right, bottom left, and top left).

Stimuli for the eye tracking task consisted of photographs of eight female and seven male identities, with each identity showing five different emotional expressions: Happy, fearful, angry, surprised, or neutral expression, resulting in overall 75 facial expressions. The five emotional expressions of one identity were presented simultaneously on five different positions (center, top right, bottom right, bottom left, top left; see Figure 1). Therefore, one stimulus consisted of five pictures of one identity, each showing one of the five emotions. There were 15 different identities and accordingly 15 stimuli were presented. The positions of the different emotional expressions were pseudo-randomized between trials, such that each emotion was shown unpredictably three times at each of the five positions within the fifteen trials. The images were chosen from the Radboud Faces Database (83) and were presented on a gray background for 15 s each. The free viewing task was developed using EyeLink Experiment Builder (SR Research Ltd., version 2.1.140).

### Apparatus

The eye tracker used was an EyeLink 1000 Plus Desktop Mount system (SR Research, Mississauga, ON, Canada). To control the eye tracker, EyeLink 1000 Plus Host software was used on a Host PC.

During stimulus presentation, the camera recorded gaze location and pupil diameter for both eyes based on the reflection of near-infrared light from the cornea and pupil. Gaze and pupil information was sampled binocular at a frequency of 1,000 Hz with a 2 mm lens and with a spatial resolution of 0.01°. System specifications included an average accuracy of 0.25–0.5 degrees of visual angle and a tolerance of head movements within a range of 22 × 22 cm. Participants were seated on a chair in front of a display screen (24 inch LCD screen monitor, resolution 1,920 × 1,080 pixels), with a distance of 90 cm from the display and 60 cm from the eye tracker, respectively. The recording was performed in remote mode, where the pupil could be tracked using a forehead “sticker” as reference point.

The associated Display PC presented the stimuli through EyeLink Experiment Builder (SR Research Ltd., version 2.1.140).

### Procedure

#### Calibration and Validation

For processing the tasks and simultaneous recording of eye movements the participants were tested individually inside a lit cabin. The luminance directly in front of the participants' eyes was measured by a digital Peaktech 5,035 light meter with a range of 0–2,000 lx (Ahrensburg, Germany) and kept constant across participants by dimming the test room light down to 70–80 lx directly in front of the participants' eyes. They were seated in an adjustable chair in front of the display screen. The eye tracker was positioned below and slightly in front of the screen.

At the beginning of the eye tracking experiment, participants completed a 13-point calibration. If the gaze accuracy was within 1° for both eyes, the gaze positions were considered as calibrated. A validation of the calibration followed. If validation was successful, the task began. If validation was unsuccessful, the eye tracker and chair were adjusted and the calibration and validation procedure was rerun until it successfully measured gaze at all locations.

Before each trial a drift correction using a central fixation point was performed to continually ensure the eye tracker was adequately tracking gaze. If the gaze accuracy was within 0.5° for both eyes drift correction was accepted. Whenever necessary, adjustments to the calibration were made.

#### Stimuli Presentation

One examiner gave the instructions while sitting next to the participant inside the cabin. A second examiner was sitting in an adjoining room and monitored eye movements to ensure that participants remained attentive and completed the tasks according to the instructions. The present free viewing task was part of a larger test battery comprising of different saccade and fixation tasks, a visual search task as well as different free viewing tasks, during a two-and a half-hours session, interleaved by three 10-min breaks. The order of the tasks was counterbalanced across participants of each group. As a reward for their participation in the study, participants could choose between cinema or book vouchers worth 7.50€ per hour.

Before the start of the task, participants were asked by standardized verbal instructions to look at the faces as they appeared on the screen and to answer a question after the presentation. Each trial began with the presentation of a written instruction on screen, to look at the faces for 15 s. The following instruction on the screen asked the participants to focus on a cross in the center of the screen. The participants had to look within an area of interest (AOI: 1.6 × 1°) around a fixation cross for at least 1,000 ms to start the trial. After that, a set of faces was shown for 15 s following a 1,000 ms inter-stimulus interval during which the screen was blank. The screen background color was kept gray during both stimuli presentation and interstimulus interval. After the presentation of each trial the instructor asked which emotions they had just seen on the screen.

### Data Analysis

The validity of the eye movement data was assessed with a proportion of missing values, which was acceptable for all groups (TD: 1.6%, SCZ: 5.2%, ADHD: 1.4%, ASD: 1.1%), albeit significantly higher in the SCZ compared to all other groups (GROUP: *F*_3, 124_ = 10.960, *p* < 0.001, η^2^ = 0.210). Data analysis was performed using the data analysis program EyeLink Data Viewer (SR Research Ltd., version 3.1.97). Any period that was no blink or saccade, was defined as “fixation” according to the proprietary analysis algorithm.

Five different AOIs of equal size, one for each face, were defined for each of the stimuli. From these, dwell time and numbers of fixations (“fixation count”) were derived for each AOI, both in absolute terms and as proportions of times participants were actually looking at the stimuli (rather than producing artifacts like blinks, or looking away from the screen). As absolute and relative measures yielded largely consistent results, we focus here on the relative measures unless conclusions to be drawn from these results conflict with each other. Such measures have been validated in previous studies to capture visual attention (62, 84–89).

A 5 × 5 × 4 mixed ANOVA with the within-subjects factors EMOTION (levels: Happy, fearful, angry, surprised, or neutral expression) and POSITION (levels: Center, top right, bottom right, bottom left, top left), and the between-subject factor GROUP (levels: ADHD, ASD, SCZ, TD) was used for each of the dependent variables. Furthermore, we ran subsequent mixed ANOVAs between each clinical group and the TD group with related contrast analyses as well as Bonferroni-adjusted *post-hoc* analyses for comparisons between groups and emotions. Additionally, we executed pairwise ANOVA's between the clinical groups. As a control analysis, the interaction between the two factors EMOTION and POSITION was examined. Further control analyses showed that neither age nor gender influenced the free viewing results significantly. Regarding gender, there was a significant chi-square test and therefore we included gender as a variable in different kinds of ANOVAs using the three groups TD, ADHD, and SCZ (in the ASD group, this was unfeasible as this group included only one female participant). We neither found any significant gender effects in these three groups analyzed separately, nor was there any significant gender ^*^ group interaction when either of the two clinical groups alone or both clinical groups together were compared with TD. Given the aforementioned gender imbalance between groups, we also explored potential gender effects of the stimulus materials by comparing dwell times and fixation counts for male vs. female faces. We found that within the contemplation period of 15 s and across all participants, female faces were looked at some 38 ms longer than male faces (*p* = 0.06, *Cohen's d* = 0.625). Neither did this effect interact with group status (TD, ASD, ADHD, SCZ) nor the subjects own gender. In order to check out the stability of our ANOVA findings for group differences in IQ, IQ was added as a covariate according to the suggestions by Schneider et al. (90), that is, after mean-centering the covariates when the design contains within-subject factors, using ANCOVA. This resulted in only negligible changes in the results presented here. Given that the correction for IQ differences is a controversial (91) and apparently not yet settled issue, we report the ANOVA results throughout.

Analyses were performed with SPSS software, Version 27 (SPSS Institute Inc., Cary, NC, USA). A significance level of α = 0.05 was adopted for all statistical analyses and partial η^2^ (η^2^) quantified effect sizes.

Given the sample size, it can be assumed that the mixed ANOVA is sufficiently robust to violations of the normal distribution (92–94). Greenhouse-Geisser adjustments were made to correct for violations of sphericity. Homogeneity of the error variances was not met for all variables, as assessed by Levene's test. There was homogeneity of covariances, as assessed by Box's test (*p* = 0.002). Overall, the robustness of the analysis with respect to the preconditions is given.

According to G^*^Power (95), a group size of at least 20 subjects per group allows us to find an effect (*f* ) of 0.25 at an alpha level of 0.05 with a test power of 0.90 (*N* = 80, *df* = 3). The final sample satisfied these requirements.

## Results

The subsequent results section is split into three major parts. In *part 1*, results on dwell time and results on fixation counts are presented. For each of these variables we found significant EMOTION ^*^ GROUP interactions suggesting group-specific processing of emotional faces. To disentangle these interactions, we first looked at the EMOTION effects for controls, followed by pairwise comparisons between controls on the one side, an each of the clinical groups on the other. In *part 2*, we compared the clinical groups (ASD, ADHD, SCZ) with each to directly address commonalities and differences. In *part 3*, we present additional, secondary findings.

### Comparisons Between the Clinical Groups on the One Side and Controls on the Other

#### Dwell Time

Mixed ANOVA for proportion of dwell time and all groups with a Greenhouse-Geisser correction revealed a significant effect of the within-subject factor EMOTION on dwell time (*F*_2, 999, 371.833_ = 8.211, *p* < 0.001, η^2^ = 0.062) which was further qualified by an overall EMOTION ^*^ GROUP interaction (*F*_8.996, 371.833_ = 2.113, *p* = 0.028, η^2^ = 0.049). This indicates that the dwell times for the different emotional expressions differed between the groups. To break down this interaction, subsequent mixed ANOVAs were conducted between each clinical group and the control group.

**Figure 2:**
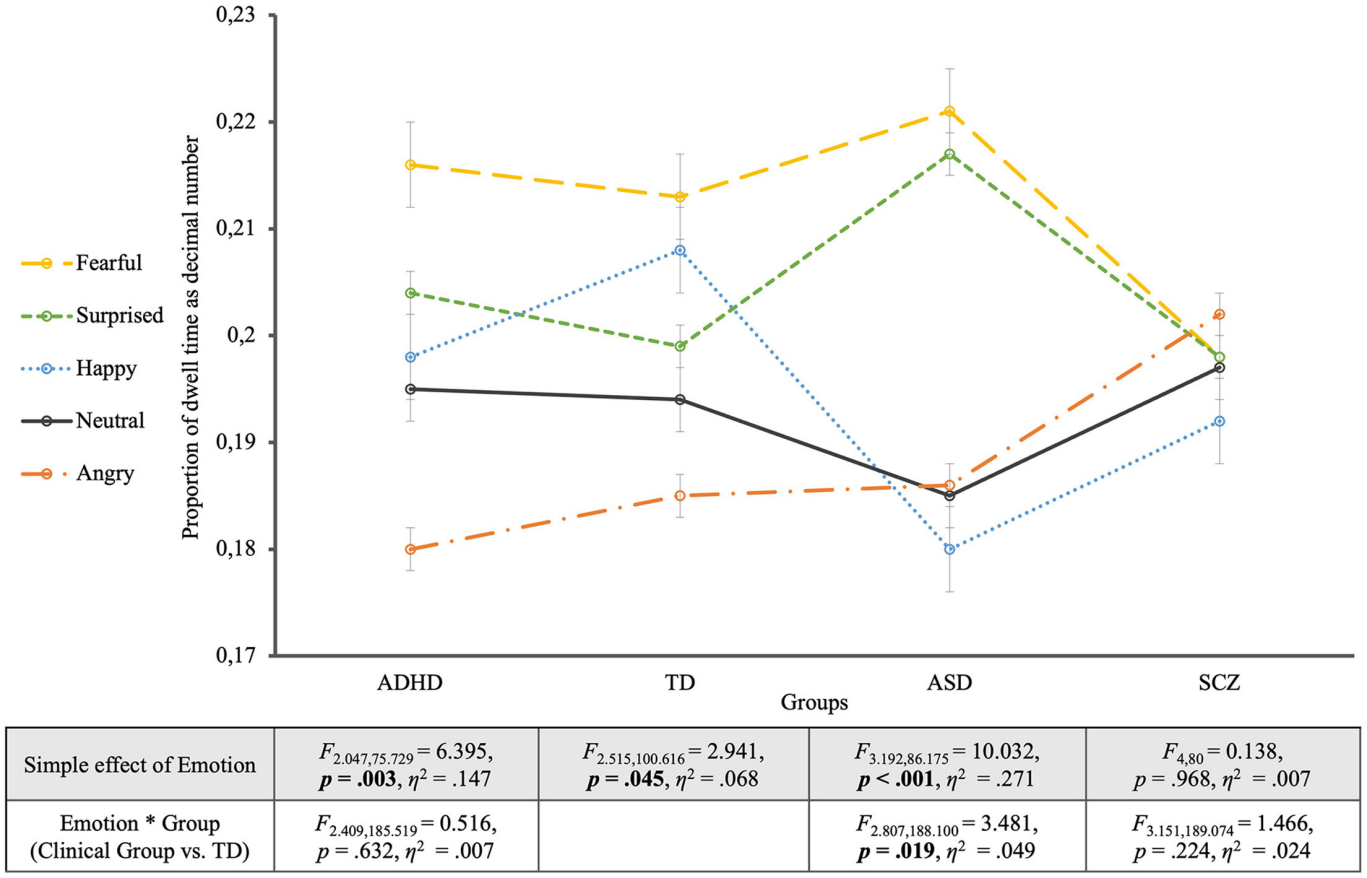
Proportion of dwell time for the four groups. The groups are located on the horizontal axis with the different emotions as single lines. The table shows the statistics of the simple effects of EMOTION as well as the interaction effects EMOTION ^*^ GROUP.

When looking at the TD group's viewing behavior only (see Figure 2), to begin with, it turned out that controls' gazes dwelled longest on fearful faces and shortest on angry faces (EMOTION: *F*_2.515, 100.616_ = 2.941, *p* = 0.045, η^2^ = 0.068). Contrast analyses between each emotional face and the neutral one revealed no significant differences though (*p* > 0.05). Otherwise, Bonferroni-adjusted *post-hoc* analysis revealed a significant difference in dwell time just between the fearful faces and the angry faces [0.028, 95% CI (0.004, 0.052), *p* = 0.011], but not between any other expressions (*p* > 0.05).

To disentangle the overall ANOVA results for all groups, “pairwise” ANOVAs comparing the TD group with one of the clinical groups were accomplished. With one exception, these ANOVAs revealed non-significant GROUP effects (TD vs. clinical group) both for the relative and the absolute dwell times. The exception was the absolute dwell time for the comparison including SCZ participants, as outlined below.

The comparison of the *ASD group* with controls showed a significant interaction between EMOTION and GROUP (*F*_2.807, 188.100_ = 3.481, *p* = 0.019, η^2^ = 0.049; EMOTION: *F*_2.807, 188.100_ = 7.977, *p* < 0.001, η^2^ = 0.106). This interaction revealed significantly longer dwell times for surprised faces and shorter ones for happy faces in the ASD group, compared to controls. Contrast analyses with the neutral expression as reference category revealed a significant interaction between EMOTION and GROUP in comparison with the surprised expression (*F*_1, 67_ = 6.878, *p* = 0.011, η^2^ = 0.093). The EMOTION effect for the ASD group alone turned out highly significant (EMOTION: *F*_3.192, 86.175_ = 10.032, *p* < 0.001, η^2^ = 0.271), showing that compared to neutral faces fearful and surprised faces were significantly longer looked at (*p* = 0.001). *Post-hoc* analyses additionally showed significantly longer dwell times for fearful and surprised faces compared to happy and angry faces (*p* < 0.05).

The mixed ANOVA between the *ADHD group* and the TD group showed differences in dwell times for the different emotions but no significant interaction with group membership (EMOTION ^*^ GROUP: *F*_2.409, 185.519_ = 0.516, *p* = 0.632, η^2^ = 0.007). For the effect of EMOTION (*F*_2.409, 185.519_ = 7.888, *p* < 0.001, η^2^ = 0.093), contrast analysis with the neutral expression as reference showed that fearful faces were significantly longer viewed (*F*_1, 77_ = 7.691, *p* = 0.007, η^2^ = 0.091) and angry faces significantly shorter than the neutral ones (*F*_1, 77_ = 7.037, *p* = 0.010, η^2^ = 0.084). Similar to controls, participants with ADHD showed an effect of the considered emotion on dwell time (EMOTION: *F*_2.047, 75.729_ = 6.395, *p* = 0.003, η^2^ = 0.147). Contrast analyses for the ADHD group showed that fearful faces were viewed significantly longer (*F*_1, 37_ = 4.874, *p* = 0.034, η^2^ = 0.116) and angry faces were viewed significantly shorter (*F*_1, 37_ = 4.727, *p* = 0.036, η^2^ = 0.113) compared to neutral faces. Furthermore, pairwise comparisons according to Bonferroni adjusted *post-hoc* analyses revealed that fearful and surprised faces were significantly longer viewed than angry faces (*p* < 0.001).

The comparison between the *SCZ group* and the TD group revealed neither a significant main effect of EMOTION on dwell time (*F*_3.151, 189.074_ = 0.728, *p* = 0.543, η^2^ = 0.012), nor a significant EMOTION ^*^ GROUP interaction (*F*_3.151, 189.074_ = 1.466, *p* = 0.224, η^2^ = 0.024). Despite the non-significance of the EMOTION ^*^ GROUP interaction, Figure 2 revealed a lack of differentiation of emotions by dwell times in the SCZ group for which not only the EMOTION simple effect was non-significant (EMOTION: *F*_4, 80_ = 0.138, *p* = 0.968, η^2^ = 0.007) but also all contrasts of the different emotions with the neutral facial expression (*p* > 0.6) as well as the *post-hoc* analyses between the different emotions (*p* > 0.05). The SCZ group was the only group that showed significantly shorter overall dwell times when compared to controls. This effect was very strong for the absolute (GROUP: *F*_1, 60_ = 44.869, *p* < 0.001, η^2^ = 0.428) and considerable weaker for the relative dwell times (GROUP: *F*_1, 60_ = 3.869, *p* = 0.054, η^2^ = 0.061) that took into account the somewhat greater proportion of missing data in this group.

To summarize the described results, three different viewing patterns have emerged for the different clinical groups: Both the ADHD and the ASD group differed descriptively from controls in the proportions of dwell times for the different emotions, but all contemplated fear longest. While the ADHD group did not differ significantly from the TD group regarding the patterns of dwell times for the different emotions, the ASD and TD groups entered a significant interaction EMOTION ^*^ GROUP showing a partially different “ranking” of dwell durations for different emotions, especially regarding the emotion of surprise and overall greater dwell time differences across emotions in ASD compared to TD. For the SCZ group, dwell times showed significantly shorter viewing times in comparison with TD (more so for absolute than relative dwell times), but also no significant interaction EMOTION ^*^ GROUP.

#### Fixation Count

Mixed ANOVA for all four groups for proportion of fixation count with a Greenhouse-Geisser correction revealed a significant effect of EMOTION (*F*_2.953, 366.179_ = 12.902, *p* < 0.001, η^2^ = 0.094) as well as for the between-subject factor GROUP (*F*_3, 124_ = 5.859, *p* < 0.001, η^2^ = 0.124), which was further qualified by an EMOTION ^*^ GROUP interaction (*F*_8.859, 366.179_ = 2.276, *p* = 0.018, η^2^ = 0.052). To describe what this interaction was due to, subsequent mixed ANOVAs were conducted between each clinical group and the control group.

**Figure 3:**
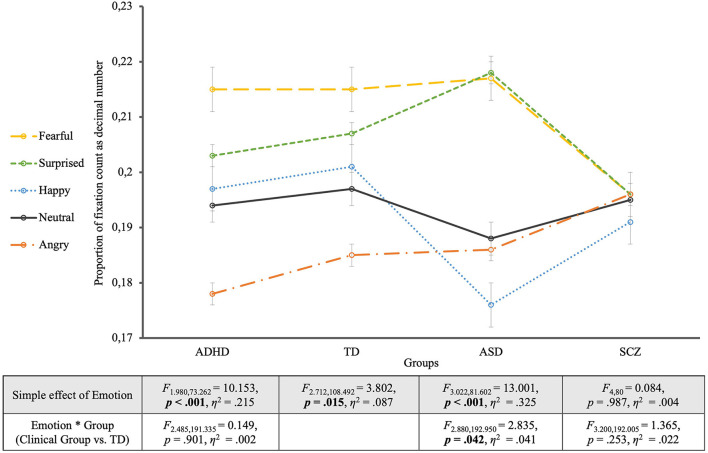
Proportion of fixation count for the four groups. The groups are located on the horizontal axis with the different emotions as single lines. The table shows the statistics of the simple effects of EMOTION as well as the interaction effects EMOTION ^*^ GROUP.

Looking at the TD group's viewing behavior alone (see Figure 3), it was shown that controls spent the most fixations regarding fearful faces and the least on angry faces (EMOTION: *F*_2.712, 108.492_ = 3.802, *p* = 0.015, η^2^ = 0.087). Contrast analyses between each emotional face and the neutral one revealed no significant differences though (*p* > 0.05). Only the comparison between the neutral face and the fearful face was on the verge of significance (*F*_1, 40_ = 4.082, *p* = 0.050, η^2^ = 0.093). Furthermore, Bonferroni-adjusted *post-hoc* analysis revealed significant more fixations for the fearful faces compared to angry faces [0.030, 95%-CI (0.009, 0.050), *p* = 0.001], and likewise for surprised faces compared to angry faces [0.022, 95%-CI (0.002, 0.042), *p* = 0.025], but no differences between any other expressions (*p-values*>0.05).

The comparison of the *ASD group* with controls revealed, additionally to the effect of GROUP (*F*_1, 67_ = 5.418, *p* = 0.023, η^2^ = 0.075) and EMOTION (*F*_2.880, 192.950_ = 11.873, *p* < 0.001, η^2^ = 0.151), a significant interaction between EMOTION and GROUP (*F*_2.880, 192.950_ = 2.835, *p* = 0.042, η^2^ = 0.041). This interaction was due to significantly more fixations for surprised faces and fewer fixations for happy faces in the ASD group, compared to controls. Contrast analyses with the neutral expression as reference category revealed a significant interaction between EMOTION and GROUP in comparison with the surprised expression (*F*_1, 67_ = 4.776, *p* = 0.032, η^2^ = 0.067). For the ASD group alone the EMOTION effect was highly significant (EMOTION: *F*_3.022, 81.602_ = 13.001, *p* < 0.001, η^2^ = 0.325), with an effect size almost four times as large as in controls (η^2^ = 0.087, see above), showing that compared to neutral faces fearful and surprised faces were significantly more often fixated (*p* < 0.003). Furthermore, *post-hoc* analyses revealed significantly more fixations for fearful and surprised faces compared to happy and angry faces (*p* < 0.05).

The mixed ANOVA between the *ADHD group* and the TD group showed an effect of the different emotions on the proportion of fixation count but no significant interaction with group membership (EMOTION ^*^ GROUP: *F*_2.485, 191.335_ = 0.149, *p* = 0.901, η^2^ = 0.002) but a generally lower proportion of fixations for the ADHD group (GROUP: *F*_1, 77_ = 6.081, *p* = 0.016, η^2^ = 0.073). For the effect of EMOTION (*F*_2.485, 191.335_ = 11.776, *p* < 0.001, η^2^ = 0.133), contrast analyses with the neutral expression as reference showed that fearful faces (*F*_1, 77_ = 11.767, *p* = 0.001, η^2^ = 0.133), and surprised faces (*F*_1, 77_ = 5.478, *p* = 0.022, η^2^ = 0.066) received significantly more, and angry faces significantly less fixations than the neutral ones (*F*_1, 77_ = 11.849, *p* = 0.001, η^2^ = 0.133). Equal to the TD group, participants with ADHD showed an effect of the considered emotion on fixation count (EMOTION: *F*_1.980, 73.262_ = 10.153, *p* < 0.001, η^2^ = 0.215). Contrast analyses for the ADHD group showed that fearful faces were fixated significantly more often (*F*_1, 37_ = 8.800, *p* = 0.005, η^2^ = 0.192) while angry faces were fixated significantly less often (*F*_1, 37_ = 11.446, *p* = 0.002, η^2^ = 0.236) compared to neutral faces. Pairwise comparisons according to Bonferroni adjusted *post-hoc* analyses revealed that fearful and surprised faces were significantly more often fixated than angry faces (*p* < 0.001).

The analysis including the *SCZ group* and the TD group revealed neither a significant main effect of EMOTION on proportion of fixation count (*F*_3.200, 192.005_ = 1.429, *p* = 0.234, η^2^ = 0.023), nor a significant EMOTION ^*^ GROUP interaction (*F*_3.200, 192.005_ = 1.365, *p* = 0.253, η^2^ = 0.022), but a main effect of GROUP (*F*_1, 60_ = 8.931, *p* = 0.004, η^2^ = 0.130), showing a significantly lower proportion of fixation count for the SCZ Group Regarding the Faces. For the SCZ Group Alone There Was a Lack of Differentiation of emotions by fixation count for which not only the EMOTION simple effect was non-significant (EMOTION: *F*_4, 80_ = 0.084, *p* = 0.987, η^2^ = 0.004) but also all contrasts of the different emotions with the neutral facial expression (*p* > 0.7).

In summary, similar results were found for fixation counts as for viewing times. The ADHD group did not differ significantly from the TD group regarding to patterns of fixation count, even showed the same “ranking” of emotions. A significant EMOTION ^*^ GROUP interaction was found only when comparing the ASD group with TD and pointed to more fixations falling on surprise in ASD compared to TD. The SCZ group again did not differentiate between emotions and again did not interact with the TD group with regard to fixation counts. Despite these differences between the clinical groups, they all showed overall a lower proportion of fixation counts compared to TD.

### Comparisons Between the Clinical Groups

#### Dwell Time

Further, we ran pairwise mixed ANOVA's between the clinical groups for *proportion of dwell times* and fixation counts (see Table 2 for all results).

**Table 2:** Pairwise mixed ANOVA's between the clinical groups.

**Dependent variable**	**ADHD–ASD**			**ADHD–SCZ**			**ASD–SCZ**		
	***F***	***p***	***η^2^***	***F***	***p***	***η^2^***	***F***	***p***	***η^2^***
**Proportion of dwell time**									
EMOTION	14.634	**<0.001**	0.186	1.689	0.170	0.029	3.904	**0.005**	0.077
EMOTION * GROUP	2.484	0.070	0.037	2.273	0.081	0.038	3.186	**0.015**	0.063
GROUP	0.528	0.470	0.008	1.931	0.170	0.033	0.123	0.728	0.003
POSITION	87.432	**<0.001**	0.577	74.417	**<0.001**	0.566	71.282	**<0.001**	0.603
POSITION * GROUP	0.905	0.385	0.014	0.578	0.535	0.010	0.025	0.958	0.001
**Proportion of fixation count**									
EMOTION	20.152	**<0.001**	0.239	3.097	**0.030**	0.052	5.458	**0.001**	0.104
EMOTION * GROUP	4.068	**0.012**	0.060	2.938	**0.037**	0.049	4.003	**0.007**	0.078
GROUP	0.162	0.689	0.003	4.538	**0.037**	0.074	2.399	0.128	0.049
POSITION	131.285	**<0.001**	0.672	105.479	**<0.001**	0.649	110.477	**<0.001**	0.702
POSITION * GROUP	1.336	0.267	0.020	2.993	0.051	0.050	0.575	0.563	0.012

*Bold values indicate significant results*.

The analysis for the *ASD and ADHD groups* for the proportion of dwell times revealed a significant effect of EMOTION (*F*_2.669, 170.826_ = 14.634, *p* < 0.001, η^2^ = 0.186) but no other significant effects or interactions (*p* > 0.05).

The analysis including the *ASD* and the *SCZ group*, by contrast showed a significant interaction between EMOTION and GROUP (*F*_4, 188_ = 3.186, *p* = 0.015, η^2^ = 0.063; EMOTION: *F*_4, 188_ = 3.904, *p* = 0.005, η^2^ = 0.077).Contrast analyses with the neutral expression as reference category revealed a significant interaction between EMOTION and GROUP in comparison with the surprised expression (*F*_1, 47_ = 4.553, *p* = 0.038, η^2^ = 0.088), showing significantly longer dwell times for surprised faces in the ASD group, compared to SCZ.

For the comparison of the *ADHD group* and the *SCZ group*, there was no significant effect of EMOTION (*F*_3.049, 173.807_ = 1.689, *p* = 0.166, η^2^ = 0.029) or GROUP (*F*_1, 57_ = 1.931, *p* = 0.170, η^2^ = 0.033) and no significant interaction (*p* > 0.05).

#### Fixation Count

The pairwise mixed ANOVAs between the clinical groups for proportion of *fixation counts* all revealed significant effects of EMOTION (2.9 ≤ *F* ≤ 4.1; 0.007 ≤ *p* ≤ 0.04) and significant interactions between EMOTION and GROUP.

Regarding *SCZ vs. ADHD* contrast analyses between the emotional faces and the neutral expression as reference showed no significant effect (*p* > 0.05).

Comparing the comparison between *SCZ* and *ASD*, our analysis showed significant more fixations for surprised faces compared to the neutral faces (*F*_1, 47_ = 6.949, *p* = 0.011, η^2^ = 0.129). The significant interaction of GROUP and EMOTION (*F*_1, 47_ = 5.889, *p* = 0.019, η^2^ = 0.111) was primarily due to the faces showing surprise and eliciting more fixations in the ASD compared to the SCZ group.

Comparisons of the *ADHD* and the *ASD group* revealed a significant GROUP ^*^ EMOTION interaction (*F*_2.504, 160.241_ = 4.068, *p* = 0.012, η^2^ = 0.060) that was nourished by more fixations for surprised, fearful and angry faces in the ASD compared to the ADHD group.

For the comparison between the *SCZ* and the *ADHD group*, there was significant effect of GROUP (*F*_1, 57_ = 4.538, *p* = 0.037, η^2^ = 0.074) on proportions of fixation count, showing more fixations for the ADHD than for the SCZ group. However, the interaction of GROUP ^*^ EMOTION turned out significant (*F*_2.890, 164.746_ = 2.938, *p* = 0.037, η^2^ = 0.049), contrast analysis between the emotional faces and the neutral expression as reference showed no significant effect (*p* > 0.05). The interaction is therefore due to more fixations for the fearful faces and less fixations to the angry faces in the ADHD group, compared to the SCZ group, which looked at both emotions equally often.

### Secondary Results—Effect of Position

As a secondary result, a significant effect of the within-subject factor POSITION was found for both the proportion of dwell time (*F*_1.754, 21.444_ = 153.779, *p* < 0.001, η^2^ = 0.554) and of number of fixations (*F*_2.382, 295.396_ = 209.528, *p* < 0.001, η^2^ = 0.628), which can be further differentiated by a POSITION ^*^ GROUP interaction.

Accordingly, dwell times and fixation counts for the different positions differed between groups and emotions. To break down these interactions, we looked at the subsequent mixed ANOVAs between each clinical group and the control group.

For all groups, there was a significant effect of POSITION, in the sense that the central position was viewed significantly longer and fixated more often than all other positions (*p* < 0.001). This “central bias” was more pronounced in the SCZ group and in the ASD group compared to TD, shown by significant POSITION ^*^ GROUP interactions (POSITION effects for: (a) dwell times—SCZ: *F*_2.039, 122.347_ = 4.947, *p* = 0.008, η^2^ = 0.076; ASD: *F*_1.806, 120.994_ = 6.218, *p* = 0.004, η^2^ = 0.085; (b) fixation counts—SCZ: *F*_2.619, 157.125_ = 6.493, *p* = 0.001, η^2^ = 0.098; ASD: *F*_2.538, 170.022_ = 4.489, *p* = 0.007, η^2^ = 0.063). Comparing the POSITION effects directly between the ASD and SCZ groups we found no significant interaction between GROUP and POSITION [(a) dwell times—*F*_1.657, 77.901_ = 0.025, *p* = 0.958, η^2^ = 0.001; (b) fixation counts—*F*_1.979, 93.035_ = 0.575, *p* = 0.563, η^2^ = 0.012; simple effects of POSITION: (a) dwell times—*F*_1.657, 77.901_ = 71.282, *p* < 0.001, η^2^ = 0.603; (b) fixation counts—*F*_1.979.93.035_ = 110.477, *p* < 0.001, η^2^ = 0.702].

## Discussion

The present study examined young adults with ASD, ADHD, or schizophrenia under the assumption of overlapping pathophysiological mechanisms, with the aim of investigating similarities and differences between these groups regarding visual exploration of emotional faces. To that end, the three clinical groups were each compared with a control group regarding the visual exploration of five different facial emotions presented simultaneously while eye fixations were recorded to analyse dwell times and fixation counts.

We found the following main results: (a) The *ASD group* differed significantly from TD in differentiating more strongly between emotions and “ranking” emotions partially differently regarding dwell times and fixation counts. (b) The TD and *ADHD groups* showed rather similar corresponding fixation patterns for the different emotions, both regarding dwell times and fixation counts. (c) The *SCZ group*, by contrast, differentiated not at all between emotions and exhibited reduced dwell times compared to controls. (d) While the ASD group and the SCZ group differed from ADHD in fixation counts and not in dwell times, regarding the attentional preferences for different emotions, dwell times differentiated the ASD and the SCZ group in that aspect, additionally to fixation counts. Furthermore, the total amount of fixations differentiated the ADHD and SCZ group.

The healthy control subjects looked at the different emotions for different lengths of time and fixated them with different frequencies. This speaks to our prediction of an influence of emotional facial expression on visual exploration behavior. In particular, this was shown to be significant for the difference between the emotions fear and anger for both viewing duration and fixation number. These facial emotional expressions had been highlighted in previous studies already. For example, Green et al. (47) found increased numbers and duration of fixations for fear and anger compared to other emotions (happy, sad, neutral). While many studies suggested that negative information and, consequently, negative emotions attract more attention than positive information (47, 96, 97), other studies showed that there are in addition different responses to different negative emotions (49, 98–100). Accordingly, the mere valence of emotions cannot fully explain the observed viewing patterns. Also, we found that that fearful faces were viewed the longest and fixated the most, while angry faces were viewed the shortest and fixated the least frequently. Within the domain of emotions with negative valence, we thus found opposing viewing patterns. Such opposing patterns of attentional preferences may reflect an threat-based attentional bias (99) as well as more avoidance tendencies on angry faces (101). Importantly, in many studies not showing such results, only one of the two negative emotions were presented, that is either fear or anger, which may have led to increased exploration of either of these facial expressions in comparison to other ones [e.g., (102, 103)].

By contrast, Mühlenbeck et al. (49) looked at both of these emotions in their study and did so within pairwise comparisons with neutral and happy faces. Similar to our results, they found the longest viewing times for fearful facial expressions and the lowest for angry ones, and accordingly argued against a general negative attention bias. While both emotions have threat connotations, they are shown in real life for different reasons and consequently require different responses (49). Fearful faces are shown as a response to a threat in the environment, this source has to be recognized and therefore attention is directed to the fearful face (104). An angry face, on the other hand, represents a direct threat from a counterpart and consequently results in avoidance behavior (105). To avoid harmful consequences, both fearful and angry faces require very specific responses compared to positive emotions, whose response behavior is more flexible (49).

Mogg et al. (106) suggested that these behavioral patterns arise from the fact that initially angry as well as fearful faces automatically attract attention, and in a later phase gaze is averted from angry faces while being maintained for fearful faces in order to determine an appropriate response. For the first phase of attentional alignment, this is consistent with Green et al. (47) idea of increased vigilance, relative to socially threatening stimuli.

For the *ASD group*, consistent with our prediction, we found emotion-specific deviations from the TD group in viewing time and fixation number. In this group, the emotion effect showed the strongest effect size and the greatest differentiation between emotions. This attentional weighting of emotions interacts with the TD's exploration behavior. This suggests that ASD subjects process emotions in different ways as suggested by the different “rankings” of emotions regarding viewing time and fixation number. This finding, hence, cannot be explained by threat-related assumptions alone. Fan et al. (59) found in their meta-analysis a small but significant effect for a bias toward threatening faces compared to happy faces in ASD participants. Likewise, ASD subjects in our study viewed fearful faces the longest, even when compared to happy faces. The finding that fear is viewed the longest, both in ASD and TD is found in a variety of studies that showed marked scanning behavior with respect to threatening facial expressions [e.g., (47, 107–109)]. However, this enhanced attention does not apply to the further threat-related emotion anger. Similar response mechanisms to TD could be hypothesized here, making angry faces more likely to be avoided, especially in more anxious subjects (101).

Atypical fixation patterns in the ASD participants of our study referred particularly to the significantly longer viewed and more frequently fixated facial expressions of surprise (and shorter viewed and less frequently fixated happy emotion expression). In line with this result, there are studies suggesting increased attentional orienting in ASD regarding the emotion of surprise (23, 110, 111), possibly in consequence of a less frequent experience and engagement with this emotion. In agreement with this reasoning is the common observation that experienced special educators avoid surprised reactions and surprising situations when working with individuals with autism, knowing that rigidity and thus little tolerance for surprises is core to their symptomatology (23). This may be due to surprise being a particularly “cognitive” emotion according to Baron-Cohen et al. (112). The notion here is that surprise differs from the other basic emotions in that it is not evoked by a situation alone, but can only be understood if the emotion-expressing subject's belief is understood. With appropriate reasoning regarding the “Theory of Mind,” individuals with autism seem to exhibit partial difficulties (113–115), which could result in an increased attentional focus regarding such surprised expressions. Another peculiarity in the eye movements in the ASD group is shown by a more pronounced central bias, i.e., the consideration of the central position, which has not been shown so far for ASD, but has been shown for SCZ (see below).

Consistent with our prediction and previous studies, relative fixation preferences as revealed by the “ranking” of facial emotions were similar between *patients with ADHD* and control subjects. Both groups paid the most attention to the fearful facial expression and the least to the angry one. While we found minor differences for dwell times on surprised and happy expressions between TD and ADHD, the two groups showed the same ranking of emotions in terms of the proportion of fixation number. The only difference between the groups was in the proportion of fixation count, with patients with ADHD showing generally fewer fixations. Nevertheless, the similarities support the assumption that in ADHD the basic emotion processing skills are intact and not core to the symptomatology (27). Overall, it can be concluded on the basis of our results that participants with ADHD have similar attentional preferences for facial emotions as TD and thus presumably intact emotion processing. It should be noted, that from a statistical point of view not rejecting the null hypothesis does not amount to accepting it. Based on the psychopathology of ADHD, which does not include affective disturbances as core symptoms, as well as the pertinent literature (see introduction), finding no differences between participants with ADHD and neurotypical controls is the expected outcome of the comparison.

For the *SCZ group*, we found no emotion-related differences in viewing times as well as in fixation numbers. Consequently, there was no differentiation based on these eye movement parameters between emotions, which is consistent with our prediction. This group, in addition, showed significantly shorter dwell times and smaller proportions of fixation numbers for the faces, when compared to the TD group. Overall, our results indicate a generally impaired and constrained visual exploration behavior.

Constrained fixation during visual exploration in participants with schizophrenia has been well-replicated (116), and “minimal scanning behavior” or “staring” has been found to be positively correlated with blunted affect [e.g., (117–119)], a negative symptom of the disorder and one of the “four As” in Bleuler's conceptualization of schizophrenia. While it seems clinically intuitive to associate reduced exploration of emotional faces with affective flattening, it has been shown in various studies that this viewing pattern seems to be independent of the (emotional) content of the displayed pictures (116), calling for a broader explanatory construct. Visual exploration requires voluntary initiation and continuation of a specific form of behavior, the re-construction of complex visual stimuli by self-controlled and selective spatial sequencing of fixations. As such, decreased visual exploration resembles at the construct level “avolition,” another negative symptom of schizophrenia, typically defined as decrease in the ability to initiate and persist in self-directed purposeful activities. While blunted affect and avolition are different facets of negative symptomatology, finding stable two-factorial solutions to self-reported symptoms that distinguish “positive” and “negative” symptoms in schizophrenia and schizotypy (120), may point to common underlying pathophysiological “mechanisms” [e.g., in fronto-temporal or cortico-basal networks, (121)]. The restricted exploration behavior is further supported by the observation of a stronger central bias in the SCZ group compared to TD, as also reported in previous studies (67, 117). It should be noted that the SCZ participants tested here were all early-onset SCZ cases, having developed psychoses before the age of 18 years. Only about 0.1–1.0% of all SCZ cases show such an early onset, putatively due to high genetic load and with an overall poor prognosis (122).

Both in the SCZ and the ASD sample we found an increased central bias, possibly pointing to a commonality between these two disorders in a general feature of visual exploration. However, as has been shown for smooth pursuit eye movements, ASD and SCZ may exhibit dissimilar neurophysiological “mechanisms” (123) underlying similar functional deficits (124). Such topics obviously should be resolved ideally in combined functional and neurophysiological studies.

## Limitations, Conclusions, and Future Directions

Several limitations of the study should be noted. First, the sample size was small, especially for the SCZ group, necessitating replication using larger groups. This is a potential limitation of the generalizability of our findings and limits the possibility of explaining within-group heterogeneity (by analyses of inter-dependencies). Second, another limitation is that all schizophrenia patients were receiving anti-psychotic medication. There is, hence, a confounder between diagnosis and medication status. For obvious ethical reasons, excluding this confounder is practically very difficult. Nevertheless, future studies measuring emotional preferences in drug naive patients could address this crucial issue. That said, other studies did either not report any relation between visual exploration and medication (65, 125, 126) or that, if anything, medication seems to “normalize” distorted processes, sliding study outcomes toward the null hypothesis rather than producing seeming “deficits” (127). Third, given the high number of different analyses between groups and emotions, the possibility of Type I error should be noted. Given the fairly “lenient” significance threshold of *p* < 0.05, it is important to consider the statistical problems of multiple tests, that is, the inflation of the alpha error. In addition of the requirements of an independent replication of our results, this problem enhances the need for discussions of effect sizes. Fourth, a further limitation of our study is the rather long testing duration, which may lead to different time-on-task effects for a given task between individuals, depending on the position of the task in test battery. While the counter-balanced task order reduces differences between tasks regarding general time-on-task effects—as well as group differences herein—, it increases within-group heterogeneity and thus the ANOVA error term. Importantly, such effects would push statistical results toward the null hypotheses and thus reduce rather than produce significant findings. Fifth, unfortunately, not all matching criteria were adequately considered. Gender in particular was not well-balanced between groups. Gender effects in various domains, for instance in face recognition (128), have been reported in the literature. We are, however, not aware of any studies reporting gender effects in emotional preferences. Therefore, we undertook several *post-hoc* analyses of gender effects within the TD, SCZ and ADHD groups as well as interaction effects of gender and group in comparisons between SCZ and ADHD on the one side and TD on the other. All these *post-hoc* analyses showed that the present paradigm did *not* unveil any significant gender effects (see methods Section). This potential threat to the internal validity of our study design had in fact no impact on our results. As a general comment, it should be noted that if gender imbalance characterizes a clinical population as is the case with all neurodevelopmental disorders, balancing gender between such clinical groups and controls impacts the external validity of a study. Moreover, previous studies showed no effect of gender on eye-movement parameters among others also in the study of emotional face processing (129–131).

Co-morbidity with other psychiatric disorders is a key feature of all neurodevelopmental disorders as the vast majority of such patients has at least one co-morbid diagnosis. Recruiting, for instance, ASD or ADHD patients without co-morbid disorder(s) is therefore not only a highly cumbersome undertaking, is also limits the generalizability of any finding seriously. We therefore decided to constrain our exclusion criteria to serious other psychiatric, neurological and medical conditions like psychosis or substance use (for the ASD and ADHD groups) epilepsy or preterm birth. Furthermore, the participants in the SCZ group had a main diagnosis of schizophrenia, schizophreniform or schizoaffective disorder. Given that all of them were early-onset SCZ cases, this sample is very rare and in addition co-morbidities are typically present [for a review see (132, 133)]. The co-morbidities could be statistically controlled for by studies of very large samples, which is very difficult to achieve. Conversely, excluding comorbid cases would render the sample non-representative of the population. Accordingly we decided not to limit generalizability by employing further exclusion criteria.

The present study also has a number of important implications. A methodological innovation of the present study that is based on Owen and O'Donovan (6) is the direct comparison of three disorders with neurodevelopmental etiology that have been grouped on the basis of qualitatively similar cognitive impairment. In our study we could show that attention for emotional expressions differed between the ASD and SCZ clinical groups compared to TD, whereas the ADHD group showed similar gaze behavior. Likewise, the ASD and SCZ differed whereas ADHD and SCZ did not. Accordingly, the ASD group showed primarily qualitative differences in attentional preferences for facial emotions, the SCZ differed mainly quantitatively from TD, and the ADHD group showed the same “ranking” of emotions as controls. Pending replication in larger samples, such different fixation patterns would suggest that attention for emotion does not tap into pathophysiological mechanism that are common to ASD, ADHD, and SCZ. Here, other processes unrelated to emotion processing may show opposite results, as recently shown by Canu et al. (134).

The approach of the present study implies support of the *National Institute of Mental Health's Research Domain Criteria* (RDoC) project to provide a framework for future research classification systems for mental disorders [see Cuthbert (135)]. This project focuses on functional dimensions of behavior as well as cognitive and affective processes as studied across the entire range of functioning and breaks up current heterogeneous disorder categories (136). The present study considered three clinical disorders in the light of Owen and O'Donovan's (6) model of a continuum of neurodevelopmental disorders.

The coexistence of commonalities and differences between ASD, ADHD, and SCZ in no way argues against the concept of the continuum, but rather for its multi-dimensionality, by means of which the disorder patterns can be adequately described with their individual as well as common impairments, but also intact functions. Thapar et al. (2) also describe the overlap of the three disorders in many areas of cognitive functioning, but still heterogeneous in terms of clinical characteristics. Accordingly, emotion processing is not equally impaired in all these disorders, but is an aspect that differentiates between them, along with similar deviations of other cognitive functions [see Canu et al. (134)]. In general, the concept of continuum should encourage to not use clinical categories too rigidly and to not assign them exclusively based on cut-off values of diagnostic instruments (2). This goal also underlies the RDoC approach. Therefore, the results presented here provide further information for the implementation of such an alternative approach to future diagnostic practice that integrates advances in genetics, neuroscience and cognitive science with the goal of more effective diagnostic and treatment (137).

As this is the first study to use eye tracking to compare emotion perception across the three clinical disorders ADHD, ASD, and SCZ, the results represent an important reference point for future research. As recommended by previous studies, we captured visual attention during emotion viewing [see Berggren et al. (45)]. However, in future research further aspects should be considered such as the use of more natural stimulus materials such as social scenes, as well as the control of facial configuration skills and implicit emotion recognition (45). Our paradigm proved to be an effective measure of attentional alignment and might allow differentiation of clinical groups based on three different eye movement patterns. Finding impairments in eye movement patterns underscores the unique contribution of this methodology to the study of cognition as well as to differential diagnosis. The results of this study highlight the usefulness and importance of a joint investigation of disorders with neurodevelopmental etiology to examine commonalities and differences in multi-dimensional variable spaces to possibly reveal common pathophysiological mechanisms.

## Data Availability Statement

The raw data supporting the conclusions of this article will be made available by the authors, without undue reservation.

## Ethics Statement

The studies involving human participants were reviewed and approved by Ethics Committee of the University of Freiburg. Written informed consent to participate in this study was provided by the participants' legal guardian/next of kin.

## Author Contributions

DC and CK: study planning. DC, MB, and CK: data collection. JB, DC, and CK: data analysis. All authors: manuscript preparation. All authors contributed to the article and approved the submitted version.

## Conflict of Interest

The authors declare that the research was conducted in the absence of any commercial or financial relationships that could be construed as a potential conflict of interest.

## Publisher's Note

All claims expressed in this article are solely those of the authors and do not necessarily represent those of their affiliated organizations, or those of the publisher, the editors and the reviewers. Any product that may be evaluated in this article, or claim that may be made by its manufacturer, is not guaranteed or endorsed by the publisher.
